# Aberrant “Barbed-Wire” Nuclear Projections of Neutrophils in Trisomy 18 (Edwards Syndrome)

**DOI:** 10.1155/2015/163857

**Published:** 2015-12-08

**Authors:** Basil M. Kahwash, Nicholas B. Nowacki, Samir B. Kahwash

**Affiliations:** ^1^Department of Medicine, University of Indiana, Indianapolis, IN 46202, USA; ^2^Department of Pathology, The Ohio State University Wexner Medical Center, Columbus, OH 43210, USA; ^3^Department of Pathology and Laboratory Medicine, Nationwide Children's Hospital, Columbus, OH 43205, USA

## Abstract

We discuss the significance of neutrophils with increased, aberrant nuclear projections mimicking “barbed-wire” in a newborn child with trisomy 18 (T18). Increased, aberrant nuclear projections have been previously reported in trisomy of the D group of chromosomes (chromosomes 13, 14, and 15), and we report similar findings in a patient with T18. The peripheral blood smear showed relative neutrophilia with the majority (37%) of neutrophils showing two or more thin, rod-shaped or spike-shaped, and often pedunculated aberrant nuclear projections. The number of projections ranged from 2 to 6 per cell, averaged 2 per affected neutrophil, and ranged in length from 0.22 *μ*m to 0.83 *μ*m. This case confirms that the morphologic finding described is not restricted to trisomy of one of the chromosomes in group D, as implied in the literature.

## 1. Introduction

Trisomy 18 (T18), also known as Edwards syndrome, typically occurs as a result of nondisjunction during meiosis and is the second most common live-born autosomal trisomy. The overall prevalence is estimated at 1/2,500–1/2,600, and the live-born prevalence is estimated between 1/6,000 and 1/8,000 [1, 2]. The syndrome is characterized by cardiac septal defects, patent ductus arteriosus, horseshoe kidneys, dysgenesis of the central nervous system, characteristic craniofacial abnormalities, and overlapping or overriding fingers, among other features. About half of live births with T18 survive past the first week of life, and an estimated 5–10% live beyond one year of life [1]. Kagan et al. [3] compared 56,000 healthy infants to 395 infants with trisomy and showed that an algorithm of maternal age, prenatal maternal serum markers, and prenatal ultrasound screens is extremely sensitive for T18 (97% detection rate), with few false positives (3.1%).

Single, and often round and bulbous, nuclear appendages of neutrophils are commonly seen in healthy females. First described in 1949 by Barr and Bertram [4] in the motor neurons of female cats and later coined “Barr bodies,” these appendages were proven to be inactivated X chromosomes [5]. Davidson and Smith [6] showed that Barr bodies appeared as “dumbbell-” shaped nuclear appendages of neutrophils, and their presence strongly correlated with the female sex. Historically, the absence of Barr bodies in oropharyngeal epithelial cells of female buccal smears suggested Turner syndrome [7] and the presence of Barr bodies in male skin keratinocytes suggested Klinefelter syndrome [8]; however, these methods are now considered obsolete.

Increased abnormal nuclear projections were first associated with trisomy of the D group of chromosomes 13, 14, and 15 by Huehns et al. in 1964 [9]. Huehns described six children, each with trisomy of one of the chromosomes in the D group, with the majority of neutrophils showing multiple (ranging between 1 and 6) abnormal nuclear projections described as extremely variable in size and shape. Subsequent electron-microscopic studies confirmed that these projections were indeed nuclear, as they consisted of nucleoplasm often forming long stalks with terminal swellings [9, 10]; however, their exact etiology remains unclear.

## 2. Case Report

**Figure 1:**
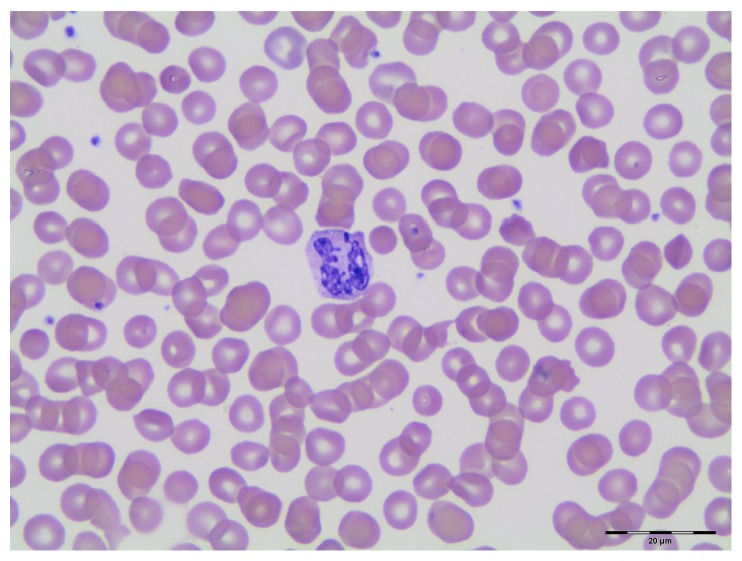
A neutrophil with aberrant thin, rod-shaped, spike-shaped, and pedunculated nuclear projections (Wright Giemsa stain, 100x oil).

**Figure 2:**
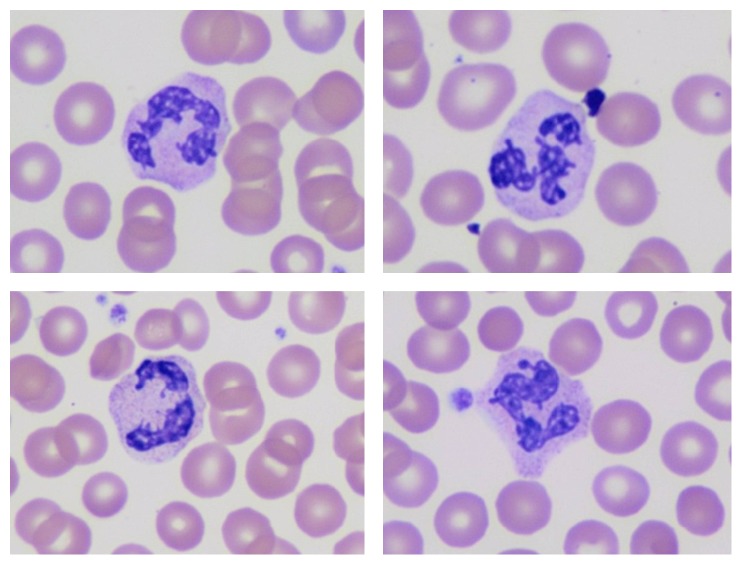
Additional neutrophils with “barbed-wire” aberrant nuclear projections (Wright Giemsa stain).

A premature, 33-week gestational age, female was noted to be cyanotic at birth and in respiratory distress. Upon further evaluation, she was also found to have multiple congenital abnormalities including bronchopulmonary dysplasia, dextrocardia, ventricular septal defect, patent ductus arteriosus, and agenesis of corpus callosum. Prenatal screening suggested T18, and it was confirmed by subsequent karyotype analysis, specifically 47,XX,+18 (10 cells counted). The patient was transferred to our tertiary care hospital at 1 month of age due to worsening respiratory function. Her initial complete blood count (CBC) showed normocytic anemia (hemoglobin 7.1 g/dL), an adequate platelet count (266 × 10^9^/L), and a normal leukocyte count (8.3 × 10^9^/L). The patient was transfused with packed red blood cells and given all needed supportive measures. A follow-up CBC showed hemoglobin of 11.7 g/dL, a platelet count of (178 × 10^9^/L), and leukocyte count (11.3 × 10^9^/L). Review of the peripheral blood smear showed a normal differential cell count with 37% of neutrophils showing two or more thin, rod- or spike-shaped, and slightly pedunculated, aberrant nuclear projections (Figures 1 and 2) mimicking the appearance of “barbed-wire.” The number of projections ranged from 1 to 6 per neutrophil, averaged 2 per cell, and ranged in length from 0.22 *μ*m to 0.83 *μ*m.

The patient's hospital stay was significant for worsening complex medical problems and deteriorating respiratory function, sadly leading to death at 3 months of age.

## 3. Discussion

**Figure 3:**
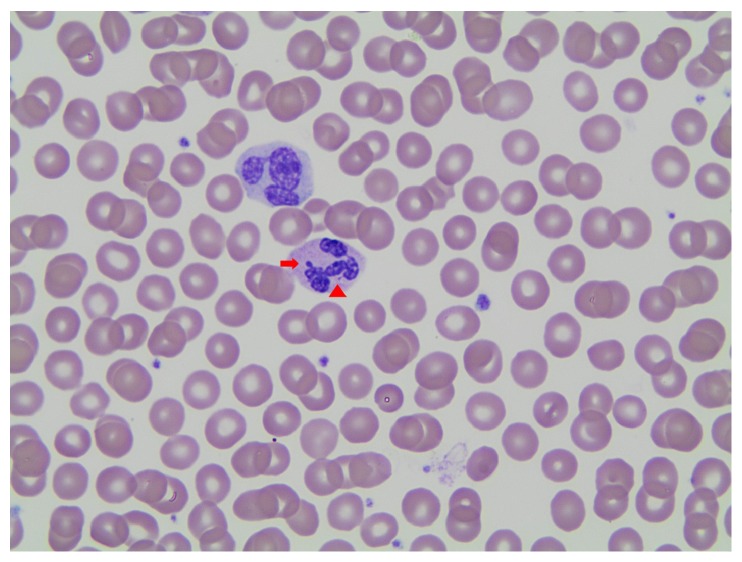
A neutrophil showing one Barr body (arrow) and one “barbed-wire” type aberrant nuclear projection (arrow head) (Wright Giemsa stain, 100x oil).

We believe that this is the first report of increased and aberrant nuclear projections in a T18 patient. Aberrant nuclear projections were first described in patients with trisomy of the D group of chromosomes 13, 14, and 15 in 1964 by Huehns et al. [9]. Later, Walzer et al. [11], comparing 11 patients with group D trisomy to 74 controls, suggested that >15% of neutrophils with two or more projections is highly suggestive of a group D trisomy. Others [10, 12, 13] reported similar findings, including one case with trisomy 14 mosaicism [14] and a single report [10] of similar findings in a patient with partial group C (chromosomes 6–12) trisomy. Across all reports, the projections were described similarly in appearance (Table 1), with a range in size of 0.25 *μ*m to 1.5 *μ*m (with Barr bodies measuring an average of 1.5 *μ*m). Aberrant nuclear projections of the “barbed-wire” type can be easily distinguished morphologically from Barr bodies. The latter tend to be single and relatively thick and show a rounded dumbbell shape with a tendency to project inwards within the nuclear curve of a neutrophil (Figure 3). We also note a single study [15] which showed similar increased aberrant nuclear neutrophilic projections in patients receiving glucocorticoids and androgen therapy.

Our case shows aberrant nuclear projections mimicking “barbed-wire” in a newborn with T18 and confirms that this morphologic finding is not restricted to trisomy of the group D/C chromosomes, as previously suggested in the literature [9–14]. Maternal screening has been shown to be effective, but not perfect, in prenatal detection of T18 and may not be available in all parts of the world. Swift interpretation, within one hour, of the peripheral blood smear by pathologists could provide additional support to a clinician's suspicion of T18 and may aid in decisions of future testing and care for these complex cases.

Finally, it should be noted that our morphologic observations and conclusions are based on a single case. Further examination and careful review of peripheral blood smears from a larger cohort of T18 patients will be helpful in further validation prior to fully incorporating this observation into the diagnostic process.

## Figures and Tables

**Table 1:** Summary of case information in articles describing aberrant nuclear projections associated with chromosomal trisomies.

Report (year)	Number of cases	Trisomy chromosome	Nuclear projections			
			Description	# per cell	Size	% of neutrophils
Huehns et al. (1964) [9]	6	D group	Extremely variable, sessile, pedunculated	1–6	0.25–1.5 *µ*m	ND
Powars et al. (1964) [12]	1	D group	Hook-like	ND	<2.0 *µ*m	63%
Lutzner and Hecht (1966) [10]	11	D group (10) C group (1)	Varied greatly in size and shape, tags, threads, clubs, drumstick-like	1–10	ND	60–80%
Walzer et al. (1966) [11]	11	D group	Variable, spherical, ovoid or club shaped	1–4	0.5–1.5 *µ*m	15–52%; Ave. = 26%
Dallapiccola et al. (1984) [14]	1	Mosaic 14	Sessile, pedunculated	3–6	0.25–1.5 *µ*m	40%
Salama et al. (2004) [13]	1	13	Thread-like	>2	ND	80%

(Ave.: average; ND: not determined).
